# Reliability and validity study of Persian modified version of MUSIC (musculoskeletal intervention center) – Norrtalje questionnaire

**DOI:** 10.1186/1471-2474-8-88

**Published:** 2007-08-31

**Authors:** Akbar Alipour, Mostafa Ghaffari, Irene Jensen, Batoul Shariati, Eva Vingard

**Affiliations:** 1 Department of Clinical Neuroscience, Section for Personal Injury, Karolinska Institutet, Sweden; 2 Department of Public Health Science, Karolinska Institutet, Sweden; 3 Department of Community Medicine, Tehran University of Medical Science, Iran; 4 Dept of Medical Sciences, Occupational and Environmental Medicine, Uppsala University, Uppsala, Sweden

## Abstract

**Background:**

Musculoskeletal disorders (MSDs) are a major health problem in the world. Self-reported questionnaires are a known method for estimating the prevalence of MSDs among the population. One of the studies concerning MSDs and their relation to work-related physical and psychosocial factors, as well as non-work-related factors, is the MUSIC-Norrtalje study in Sweden. In this study, the research group developed a questionnaire, which has been validated during its development process and is now considered a well-known instrument. The aim of this study is to validate the Persian version of this questionnaire.

**Methods:**

The first step was to establish two expert panel groups in Iran and Sweden. The Focus Group Discussion (FGD) method was used to detect questionnaire face and content validity. To detect questionnaire reliability, we used the test-retest method.

**Results:**

Except for two items, all other questions that respondents had problems with in the focus group (20 of 297), had unclear translations; the ambiguity was related to the stem of the questions and the predicted answers were clear for the participants. The concepts of 'household/spare time' and 'physical activity in the workplace' were not understood by the participants of FGD; this has been solved by adding further descriptions to these phrases in the translation. In the test-retest study, the reliability coefficient was relatively high in most items (only 5 items out of 297 had an ICC or kappa below 0.7).

**Conclusion:**

The findings from the present study provide evidence that the Persian version of the MUSIC questionnaire is a reliable and valid instrument.

## Background

Musculoskeletal disorders (MSDs) are a major health problem in all countries [1]. They encompass a variety of conditions, including disorders of muscles, tendons and nerves. Although the underlying pathology of these conditions may differ and their diagnoses are unclear, the symptoms are often similar.

There is no "golden standard" measurement tool for estimating the prevalence of MSDs among the population. Statistics on the prevalence of work-related musculoskeletal disorders may vary from one reference source to another, primarily due to variations in outcome measures, and the diagnostic criteria. A common method for estimating the magnitude of the problem is self-reported data in questionnaires concerning episodes of pain [2].

Physical, organizational, psychosocial, and sociological factors at work can contribute to MSDs alone or in combination [3-5].

The MUSIC-Norrtalje study from Sweden was performed to find health and risk factors for low back and neck/shoulder disorders in the general working population. In this study a questionnaire was designed to measure different outcomes of low back and neck/shoulder pain, as well as work-life exposure, lifestyle factors, social exposures co morbidity, life events and psychosomatic complaints [6,7]. The evolution of the questionnaire and its reliability and validity has been studied and published in Sweden [8]. This questionnaire is the combination of questions and indexes tested and approved in other studies [9-13].

In Iran there is no epidemiological information about MSDs. However, on the basis of some informal reports and statistics it seems that these are among the most frequent work-related disorders.

To generate knowledge about MSDs and to meet the increasing demand for questionnaires in Iran, we decided to translate the MUSIC questionnaire into the Persian language and validate it in a large worker population in Iran Khodro Car Manufacturing Company.

## Methods

Because of the multifactorial dimension of MSDs and the complexity of interplaying physical, psychosocial and lifestyle factors, we used the expert panel method. We established two expert groups (one in Sweden and one in Iran) from different areas including occupational medicine, epidemiology and psychology. Participants of the panel in Sweden included: one professor in occupational health (MD and PhD), one professor of psychology (PhD) and two Iranian physicians (MD and PhD student).

The panel in Iran included: two physicians, three psychologists (two at the master's and one at the PhD level), and one epidemiologist. The two groups communicated by email, and two members who were in both groups had regular meetings with the other members in Iran and Sweden.

**Figure 1:**
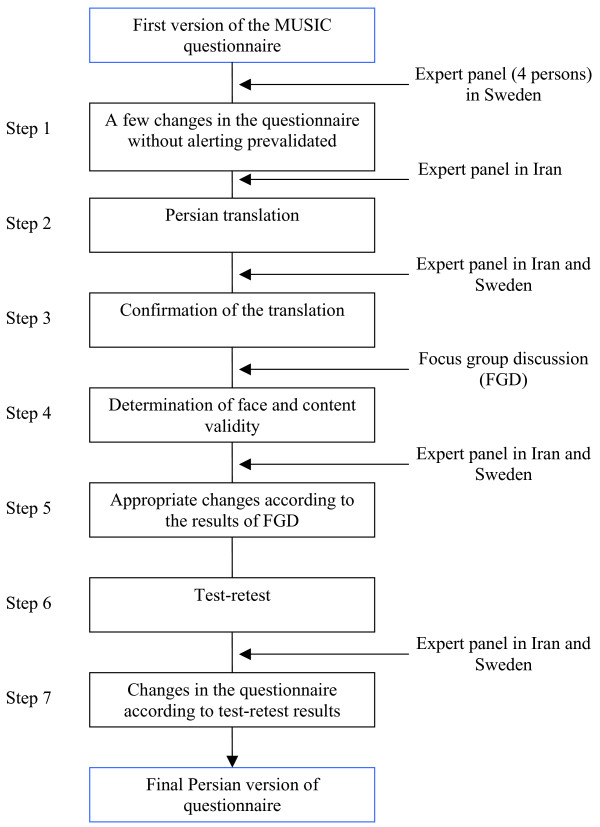
MUSIC questionnaire validity and reliability steps.

In the first step (figure 1), based on expert panel discussion, a number of changes relating to Iranian culture, word flow and related issues were made.

The English version of the modified MUSIC questionnaire we worked on included 10 domains, each consisting of a few scales with a numbers of items:

1- Demographic data (9 items)

2- General working conditions including extra work (14 items)

3- General health (18 items)

4- Sleep and recovery (24 items)

5- Musculoskeletal problems, with the scales of pain, disability, previous pain history, and clinical signs (5 scales, 129 items)

6- Working conditions, with the scales of physical working conditions, psychosocial working conditions and reorganization. In this domain, each scale consists of a few subscales with the items in it. In general, physical working conditions consists of 12 items, psychosocial working conditions 44 items, and reorganization 6 items.

7- Household/spare time (2 items)

8- Lifestyle factors (2 scales and 5 items)

9- Psychosomatic factors (17 items)

10- Life events (17 items)

In the second step we translated the questionnaire into the Persian language. The expert panel in Iran discussed the pitfalls of the translated version. This group shared their results with Swedish expert panel group. Finally in step three, both groups confirmed the translated version. Within these groups we used a consensus-building strategy that satisfied all of the members' concerns based on the disciplines they represented.

These group changes were mainly demographic ones such as: accommodation, commutation time, history of participation in the war with Iraq, and disability related to the war.

Thus the Persian version of the final questionnaire was prepared, and ready for work on its validity and reliability.

### Validity detection

In the fourth step, we used the Focus Group Discussion (FGD) method to detect questionnaire face and content validity [14].

We conducted 3 discussion groups; each group consisted of 5–6 participants with different job titles including workers, office workers, expert workers (technicians and engineers) and managers.

The main objective of the group meetings was to identify:

a- that people understood the concept of the questions.

b- that people understood the questions in the same way as the investigators did.

Each meeting lasted for 2–3 hours. One occupational physician and one psychologist were the fixed interviewers, and one psychologist or industrial nurse took it in turns to accompany them.

The focus group discussions were taped and noted. The questions discussed were:

1- Which question is ambiguous in each domain and how many participants agree with this?

2- Is ambiguity related to the question stem, or answer, or both?

3- How can the above question be changed to make it clearer?

4-Are there any other suggestions to improve the questionnaire?

### Reliability detection

To detect the reliability of the questionnaire, we used the test-retest method. 40 participants selected randomly and proportional to their job titles and levels of education (secondary school to master's level) were asked to fill in the questionnaire at 3 week intervals. Interclass Correlation Coefficients (ICCs) for the rating scale, and kappa coefficients for dichotomous answers and categorical data, were used for analyses [15].

To assess/rate the ICCs or Kappa we used the following scoring system:

>0.9 excellent

>0.8 good

>0.7 acceptable

>0.6 questionable

>0.5 poor

<0.5 unacceptable [16,17]

We analyzed all of the questions (items) in referred groups separately and one by one, but due to the large number of questions we reported the results based on their pertinent domain.

## Results

### Validity

Totally 16 people participated in 3 focus group discussion meetings. The MUSIC questionnaire consisted of 10 domains and 14 sub-domains (Scales). The total number of items in each domain and the items where there was ambiguity are shown in table 1. Out of 297 items in all, 20 items (in 7 sub-domains) were ambiguous. All of the ambiguities were related to the stem of the questions, and the predicted answers were clear for the participants. Table 2 shows ambiguous items and their frequency in each domain declared by FGD participants and the final decision made on them.

**Table 1:** Basic structure of questionnaire and validity results

Domains and Scales	Total number of items in each domain or scale	No. of items that were ambiguous in each domain	Source of ambiguity		
			
			Stem	Answer	Both
1. Demographic data	9	0			
2. General working conditions including extra work	14	0			
3. General health	18	2	*		
4. Stress, sleep and recovery	24	2	*		
5. Musculoskeletal health (in 5 body regions)	129	0			
6. Working conditions					
a. Physical	12	1	*		
b. Psychosocial	44	9	*		
c. Reorganization	6	1	*		
7. Household/Spare time	2	2	*		
8. Lifestyle					
a. Exercise	2	1	*		
b. Smoking	3	0			
9. Psychosomatic factors	17	2	*		
10. Life events	17	0			

**Table 2:** Ambiguous items and their frequency in each domain based on Focus Group Discussion

Domain	Scale	Item	Participants declaring the item to be ambiguous		Final decision
			No.	%	
General Health	_________	-I seem to get ill a little easier than other people	2	12.5	Make changes in translation
		-I expect my health to get worse	3	18.8	
Stress, sleep and recovery	_________	- Do you feel recovered and alert when you start a new work shift	8	50	Make changes in translation
		- Do you feel recovered and alert when you start working after some days off work	3	18.8	
Working conditions	Physical	-Recalling events from 5 years ago	3	18.8	Drop
	Psychosocial	-Do you think that your work tasks in your current job are stimulating	12	75	Make changes in translation
		-Does your job require too large a work effort	4	25	
		-Do conflicting demands often occur in your job	3	18.8	
		-Are you satisfied with the quality of your work	4	25	
		-Are you satisfied with your ability to cope in a positive way with your workmates	4	25	
		-Do you feel that your work performance is appreciated by your manager	3	18.8	
		-Do you experience a good and well functioning leadership from your closest manager	4	25	
		-Do you experience good and well functioning leadership from the top management	6	37.5	
		-At work, do you have access to internal training that you can participate in	4	25	
	Reorganization	-Have any of your work colleagues been under notice or been given notice of redundancy at your workplace during the last year due to reductions in work	1	6.3	Make changes in translation
Household/Spare time	_________	-How large a part of your spare time do/did you devote to housework	12	75	Make changes in translation
		- How large a part of your spare time can/could you devote to your own relaxation	12	75	
Lifestyle	Exercise	-Regular modest exercise	12	75	Make changes in translation
Psychosomatic factors		-Bad appetite	12	75	Make changes in translation
		-Hunger pangs	4	25	

Demographic data, General working condition, Musculoskeletal health, Smoking in lifestyle and life events have not had any ambiguity and were deleted in table.

In spite of the clarity of two following questions (in work-related psychosocial factors), 63% expressed concern to response to each one, although their stems were clear.

"- How many people have you seen being bullied during the last six months?

- Have you been subjected to bullying at the workplace during the last six months?"

Finally, 7 of 16 participants in Focus Group Discussion meetings felt that the number of questions in the questionnaire is large and should be reduced, and 8 (50%) people did not agree to give their names on the questionnaire sheet.

### Reliability

As questions about **demographic and general working conditions **were considered as facts and consistent with time, we did not determine their reliability.

In the **general health, sleep and recovery **domains the ICCs or kappa were more than 0.7 (acceptable).

In the **musculoskeletal **domain, the level of ICC or kappa was good and excellent (>= 0.8) in all body regions.

In the **physical working conditions **scale, the range of ICCs or kappa related to each question varied from 0.3 to 0.9. In spite of this wide range, only one coefficient was considered unacceptable/poor.

In the **psychosocial working conditions **scale, the ICCs or kappa ranged from 0.2 to 0.9.

The unacceptable/poor coefficient was related to only one question.

Regarding dichotomous questions, all kappa coefficients were significant. Thus there was good agreement in test-retest answers.

In the **reorganization **scale, there were significant coefficients in all related items.

There were two questions in the **household/spare time **domain. The first one showed an acceptable reliability coefficient and the second was questionable.

In the **lifestyle **domain, one item in the exercise scale showed a questionable reliability coefficient.

In the **psychosomatic **domain, there were excellent reliability coefficients.

In the **life events **domain, all but one question showed a significant coefficient (Table 3).

**Table 3:** Reliability test results

Domain	Scale	Range of ICC (or kappa)	Number of questions where ICC (or kappa) was above 0.7	Number of questions where ICC (or kappa) was below 0.7	The questions where ICC (or kappa) was below 0.7
General health	-	>0.7	All questions	-	-
Stress, sleep and recovery	-	>0.7	All questions	-	-
Musculoskeletal health (in 5 body regions)	-	>= 0.8	All questions	-	-
Working conditions	Physical	0.3–0.9	11	1	In your work, do/did you have to carry out the same hand or finger movements a number of times each hour? (inscribing machinery, sorting)
	Psychosocial	0.2–0.9	43	1	Do you feel that your work performance is appreciated by your customers/clients
	Reorganization	>0.7	All questions	-	-
Household/Spare time	-	0.6–0.7	1	1	How large a part of your spare time can/could you devote to your own relaxation
Lifestyle	Exercise	0.6–0.7	1	1	Regular modest exercise
	Smoking	>0.7	All questions	-	-
Psychosomatic factors	-	>0.9	All questions	-	-
Life events	-	0.6-0.9	All except one(16)	1	Illness/accident of wife/husband

## Discussion

There is no "gold standard" measurement tool for estimating the prevalence of MSDs.

Although self-reporting is usually considered a less reliable way to measure disease outcomes, MSDs is a mainly self-reported condition.

Pain is described in different ways and is of a complex nature; it is influenced by physical and psychosocial exposures, individual factors, personality and earlier experiences.

Depending on the tools (sickness absence registration, physical examination, used to measure outcome and type of complaint (self- reported pain or self-reported disabling pain), and the region of pain, the prevalence of MSDs varies widely.

Exposure assessments are another problem with regard to MSDs. For some physical and ergonomics factors, direct measurements and observations can be made. However, these are expensive and time-consuming methods. In epidemiological studies, a method that is often used for estimating the magnitude of the exposures is self-administrated questionnaires. The validity and reliability of these methods has been compared in different studies [18-22].

For psychosocial exposures, external observations are harder to perform. The experience of the worker may be more relevant when examining the relationship between exposure and outcome.

The lack of standardized exposure tools for assessing psychosocial risk factors that are relevant to work-related MSDs is considerable [23].

In Sweden, the MUSIC questionnaire has been used as a tool to study the relationship between MSDs and work-related factors. This questionnaire has been validated during its development process; it has been used in numerous publications and is considered a valid and relevant instrument.

In our study, the Persian version of the MUSIC questionnaire was developed and its validity and reliability were determined and described. It is necessary for study designers to consider features which improve subjects' reporting accuracy, including using familiar terms that are common in worksite discourse, and presenting guidelines which will help them to place their exposure in relation to that of others [24]. We used the above recommendations while translating the MUSIC questionnaire into the Persian language and in the expert panel method.

In the validity study using the Focus Group Discussion (FGD) method, we found that, only 22 out of 297 questions were ambiguous. Of them, except for two questions, all others (20) had unclear translation. For instance, after translation, the FGD participants did not understand the concept of household/spare time, so we added a description to clarify this in the translation.

The participants also mostly thought that physical activity in the workplace meant a kind of exercise. In the translated version we tried to make clear the distinction between physical exercise and physical work exposures. After this, none of the participants had any problems with the concept of the question.

Regarding company organizational culture, two questions related to work psychosocial factors (questions about bullying) were considered not to be applicable in the Persian version and were thus dropped.

Although in the original MUSIC questionnaire some questions asked about preceding exposures 20 years back in time, we restricted the time frame of the Persian version of the questionnaire to one year back in time. The reason for this was that the employees are mostly young people with shorter work experience and, if applicable, high probability of recall bias (the results of the validity study confirmed this).

In the test-retest study, the reliability coefficient was relatively high in most items, and only 5 questions out of 297 had an ICC below 0.7 (table 3).

These questions with low ICCs or kappa (only 5 questions) were dropped using expert opinion. We recommend other investigators to consider these results in their own research.

ICC is the ratio of the between-subjects variance divided by total variance [25]. It is a measure of relative reliability and in some instances can produce misleadingly high levels of reliability (for example if there is a large variance between subjects) [26]. Some researchers advise to report ICC with other measurements like SEM (standard error of measurement).

The magnitude of the Kappa coefficient represents the proportion of agreement greater than that expected by chance but there are other factors that can influence the magnitude of kappa like prevalence, bias, and non-independence of ratings [27].

MUSIC questionnaire is an expanded questionnaire with different sections. Deleting one domain (scale) or sub domain does not affect the validity of questionnaire and it depends on research group and the aims of using questionnaire. On the other hand, as it was referred questionnaire is about different regions of body (129 questions). Usually in practice we use restricted part of body region and it decrease the number of questionnaire.

In MUSIC questionnaire in general health domain we had 18 questions. For decreasing the total number of questions this domain can be substitute by GHQ-12 that other group in Iran has studied its validity and reliability [28].

Memory is an unavoidable problem in re-test situations. Subjects may remember how they answered questions and attempt to reproduce those answers during re-test. A3-week interval between tests was chosen, in part to minimize overestimate and underestimate of reliability (due to influence of memory or actual change in work condition). Based on research group opinion and human resource department, there were no modifications in job tasks, any new intervention, organizational changes, or production demands during test re-test period.

In general, the results show that the Persian version of the questionnaire has a good conceptual structure and provides reliable information on workplace factors. This questionnaire could be considered a valuable and specific instrument to assess self-reported musculoskeletal pain and work-related physical and psychosocial exposures, as well as lifestyle factors.

## Conclusion

In conclusion, the findings from the present study provide evidence that the Persian version of the MUSIC questionnaire is a reliable and valid instrument for measuring musculoskeletal pain and disorders, as well as work-related physical and psychosocial exposures and also non-work-related factors.

The main problem was word flow in the translation and a few questions that participants were uncomfortable about answering. These problems were solved during the validity study.

## Competing interests

The author(s) declare that they have no competing interests.

## Authors' contributions

Alipour A involved in study design, data collection, data analysis and writing and revising the manuscript, Vingard E and Shariati B involved in study design, data analysis and writing and revising the manuscript. Jensen I, involved in study design, writing and revising the manuscript. Ghaffari M involved in study design and writing the manuscript. All authors read and approved this manuscript.

## Pre-publication history

The pre-publication history for this paper can be accessed here:



## References

[B1] Kilbom S, Armstrong T, Buckle P, Buckle P, Fine L, Hagberg M, Haring-Sweeney M, Martin B, Punnett L, Silverstein B, Sjogaard G, Theorell T, Viikari-Juntura E (1996). Musculoskeletal disorders: Work related risk factors and prevention. Int J Occup Environ Health.

[B2] Salerno DF, Franzblau A, Armstrong TJ, Werner RA, Becker M (2001). Test-retest reliability of the upper extremity questionnaire among keyboard operators. Am J Ind Med.

[B3] Vingard E, Nachemson ALF (2000). Neck and back pain, the scientific evidence of causes, diagnosis, and treatment.

[B4] Kilbom A (1994). Repetitive work of the upper extremity :II. The scientific bases (knowledge base) for the guide. International Journal of Industrial Ergonomics.

[B5] Linton SJ (1990). Risk factors for neck and back pain in a working population in Sweden. Work stress.

[B6] Vingård E, Alfredsson L, Hagberg M, Kilbom Å, Theorell T, Waldenström M, Wigaeus Hjelm E, Wiktorin C, Hogstedt C (2000). To what extent do current and past physical and psychosocial occupational factors explain care-seeking for low back pain in a working population? Results from the Musculoskeletal Intervention Center-Norrtälje Study. Spine.

[B7] Wigaeus Tornqvist E, Kilbom Å, Vingård E, Alfredsson L, Hagberg M, Theorell T, Waldenström M, Wiktorin C, Hogstedt C (2001). The influence on neck and shoulder disorders from work-related physical and psychosocial exposure among men and women in a Swedish general population – results from the MUSIC-Norrtälje Study. Epidemiology.

[B8] Hagberg M, Hogstedt C, editors (1993). Evaluation of methods for assessment of health and exposures in epidemiological studies of musculoskeletal disorders Stockholm MUSIC Books.

[B9] Margareta Dallner (2000). Validation of the General Nordic Questionnaire for psychological and social factors at work.

[B10] Torgén M, Alfredsson L, Köster M, Wiktorin C, Smith K, Kilbom Å (1997). Reproducibility of a questionnaire for assessment of present and past physical activities. Int Arch Occup Environ Health.

[B11] Karasek RA (1979). Job demands, job decision latitude, and mental strain: Implications for job redesign. Admin Sci Quart.

[B12] Karasek R, Theorell T (1990). Healthy Work.

[B13] Toomingas A, Theorell T, Michélsen H, Nordemar R, Stockholm MUSIC 1 Study Group (1997). Association between self-rated psychosocial work conditions and musculoskeletal symptoms and signs. Scand J Work Environ Health.

[B14] Krueger RA, Casey MA (2000). Focus Groups: A Practical Guide for Applied Research.

[B15] Moyses Szklo, Javier Nieto F (2000). Epidemiology beyond the basics.

[B16] Darren G, Mallery P (1995). Spss/Pct step by step: A simple guide and reference.

[B17] Wingo PA, Higgins JE, Rubin GL, Zahniser SC (1991). An epidemiologic approach to reproductive health.

[B18] Tielemans E, Heederik D, Burdorf A, Vermeulen R, Veulemans H, Kromhout H, Hartog K (1999). Assessment of occupational exposures in a general population: comparison of different methods. Occup Environ Med.

[B19] Kromhout H, Oostendorp Y, Heederik D, Boleij JS (1987). Agreement between qualitative exposure estimates and quantitative exposure measurements. Am J Ind Med.

[B20] Ahlborg GA (1990). Validity of exposure data obtained byquestionnaire. Two examples from occupational reproductive studies. Scand J Work Environ Health.

[B21] Teschke K, Kennedy SM, Olshan AF (1994). Effect of differentquestionnaire formats on reporting of occupational exposures. Am J Ind Med.

[B22] Nordstrom DL, Vierkant RA, Layde PM, Smith MJ (1998). Comparison of self-reported and expert-observed physical activities at work in a general population. Am J Ind Med.

[B23] Rugulies R, Braff J, Frank JW, Aust B, Gillen M, Yen IH, Bhatia R, Ames G, Gordon DR, Janowitz I, Oman D, Jacobs BP, Blanc P (2004). The psychosocial work environment and musculoskeletal disorders: Design of a comprehensive interviewer-administered questionnaire. Am J Ind Med.

[B24] Teschke K, Olshan AF, Daniels JL, De Roos AJ, Parks CG, Schulz M, Vaughan TL (2002). Occupational exposure assessment in case-control studies: opportunities for improvement. Occup Environ Med.

[B25] Denegar C, Ball D (1993). Assessing reliability and precision of measurement: An introduction to intraclass correlation and standard error of measurements. Journal of sport rehabilitation.

[B26] Keating J (1998). Unreliable inferences from reliable measurements. The Australian Journal of Physiotherapy.

[B27] Sim J, Wright CC (2005). The kappa statistic inreliability studies: Use, interpretation and sample size requirements. Physical therapy.

[B28] Montazeri A, Harirchi AM, Shariati M, Garmaroudi G, Ebadi M, Fateh A (2003). The 12-item general health questionnaire (GHQ-12): translation and validation study of the Iranian version. Health Qual Life Outcomes.

